# Comprehensive genomic profiling of pediatric peritoneal mesothelioma: case report with a literature review

**DOI:** 10.1093/jscr/rjae324

**Published:** 2024-05-18

**Authors:** Jasmina Redzepagic, Zlatan Zvizdic, Nurija Bilalovic, Emir Milisic, Melika Bukvic, Semir Vranic

**Affiliations:** Department of Pathology, University Clinical Center Sarajevo, 71000 Sarajevo, Bosnia and Herzegovina; Department of Pediatric Surgery, University Clinical Center Sarajevo, 71000 Sarajevo, Bosnia and Herzegovina; Department of Pathology, University Clinical Center Sarajevo, 71000 Sarajevo, Bosnia and Herzegovina; Department of Pediatric Surgery, University Clinical Center Sarajevo, 71000 Sarajevo, Bosnia and Herzegovina; Department of Radiology, University Clinical Center Sarajevo, 71000 Sarajevo, Bosnia and Herzegovina; College of Medicine, QU Health, Qatar University, 2713 Doha, Qatar

**Keywords:** peritoneal mesothelioma, children, prognosis, genomic alterations, outcome

## Abstract

Malignant peritoneal mesothelioma is an extremely rare and poorly recognized neoplasm in children. A 5-year-old boy presented with a 1-year history of progressive painless abdominal distension. A CT revealed a 19 × 19 × 11 cm^3^ cystic mass in the right hemiabdomen, without infiltrating the surrounding structures. The tumor was completely removed by surgery. The microscopic and immunohistochemical analyses confirmed peritoneal mesothelioma. Comprehensive genomic profiling revealed no major driving mutations including *BAP1*, no fusions, but with amplifications of *AURKA, AURKC, HLA-1B, ZNF-217, OR5F1* and *MEN1* genes. Imaging follow-up 3 months after surgery revealed metastatic disease. The patient died of pneumonia at another hospital shortly after the last follow-up examination at our institution. Pediatric peritoneal mesothelioma is an extremely rare malignancy with limited targeted options and a poor prognosis. Some of the identified molecular genomic biomarkers require further exploration and validation in this cancer.

## Introduction

Malignant mesothelioma is an aggressive malignancy, arising from mesothelial cells of peritoneal, pleural, pericardial and tunica vaginalis lining cells [1]. Malignant mesothelioma is an extremely rare tumor in the pediatric population. Its incidence is estimated to be around 0.5–1.0 cases per 10 million per year [2, 3]. It is associated with a poor prognosis with an estimated median survival of only 6–12 months [1]. Pleural mesotheliomas mostly arise in patients with a history of asbestos exposure, whereas the association with asbestos exposure and other carcinogens in peritoneal mesothelioma is less clear, including pediatric peritoneal mesotheliomas, suggesting that genetic alterations could play an important role in their development and progression [4, 5].

Given their poor prognosis and lack of well-established risk factors and treatment options, we attempted to seek driver genetic alterations that define peritoneal mesothelioma and to potentially identify targetable genetic alterations to improve the outcome through next-generation sequencing.

## Case report

**Figure 1:**
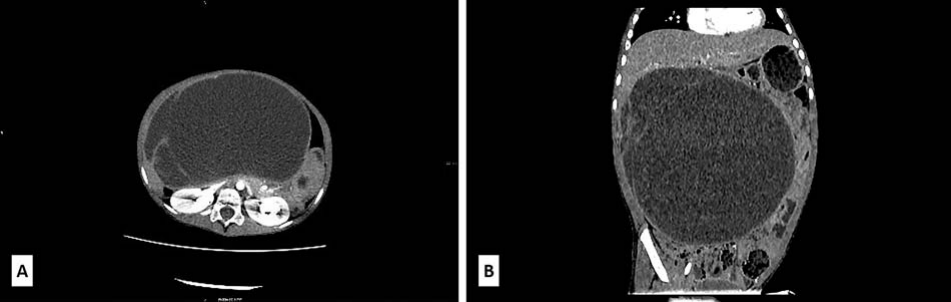
(A) Axial low-dose CT scan shows a huge cystic mass with few enhanced thin septa in the lateral aspect. Cystic space contains fluid of density 47−60HU. (B) Sagittal low-dose CT scan revealed that the cystic mass had expansive features with its cranial aspect not differentiated from the lower liver surface. No certain CT signs of liver infiltration were seen.

A 5-year-old boy presented with a 1-year history of abdominal discomfort and distension. Clinical examination by inspection and palpation revealed the presence of an abdominal mass that was confirmed by a CT scan. It revealed a multilocular cystic mass, with septations, filled with dense content (47−60 HU) and measured 113 × 190 × 195 mm^3^ (Fig. 1A). The lesion had an expansive growth but without clear liver infiltration (Fig. 1B). Differential diagnoses included mucinous cystadenoma, mesenteric lymphangioma, and hydatid cyst. A lung CT scan revealed a subpleural node in the right lower lobe, measuring 3.6 mm. Blood count test revealed elevated LDH levels of 391 U/L (range 100–190) and slightly elevated NSE level of 45.1 ng/mL (range 0–16.3) and CA 19–9 39.9 U/L (range 0–37), with normal levels of ferritin, AFP, CEA and β-HCG.

**Figure 2:**
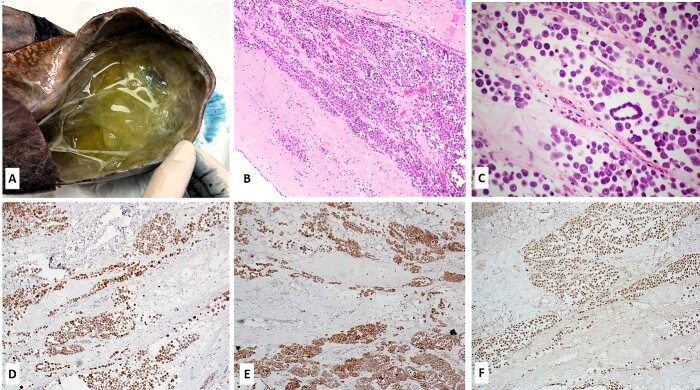
Gross, microscopic and immunohistochemical findings of the tumor. (A) Grossly, a multilocular cystic mass was seen, with cysts filled with yellowish-to-green fluid; (B–C) Microscopic features of the tumor (Hematoxylin and Eosin stain, 10X magnification image B and 20X magnification image C); the tumor cells were diffusely positive for calretinin (D), D2.40 (podoplanin) (E) and retained BAP1 protein expression (F) (immunohistochemistry, DAB staining).

The patient underwent surgery and the tumor was completely removed. Grossly, it measured 190 × 190 × 110 mm^3^, showing a multilocular cystic pattern, with cysts filled with yellowish-to-green fluid (Fig. 2A). Microscopically, the neoplasm was composed of the sheets, nests and micropapillary configurations of epithelioid cells within the loose fibromyxoid stroma (Fig. 2B). The epithelioid cells had eosinophilic cytoplasm, opened chromatin, prominent nucleoli and a few with multinucleations (Fig. 2C). Epithelioid cells were cohesive, and showed intercellular ‘windows’. Nuclear atypia was marked (Fig. 2C). The tumor cells were positive for wide-spectrum cytokeratins (AE1/AE3), CK19, calretinin, WT-1, podoplanin (D2–40) (Fig. 2D−E), while CK7, HepPar1, SALL4 and CD99 were negative. The tumor cells also retained BAP1 protein expression (Fig. 2F). Morphologic and immunohistochemical findings were consistent with peritoneal mesothelioma. Due to its rarity and challenging diagnosis, the case was also sent for a second opinion, and expert pathologists at another institutions concurred with our diagnosis (see Acknowledgement).

**Figure 3:**
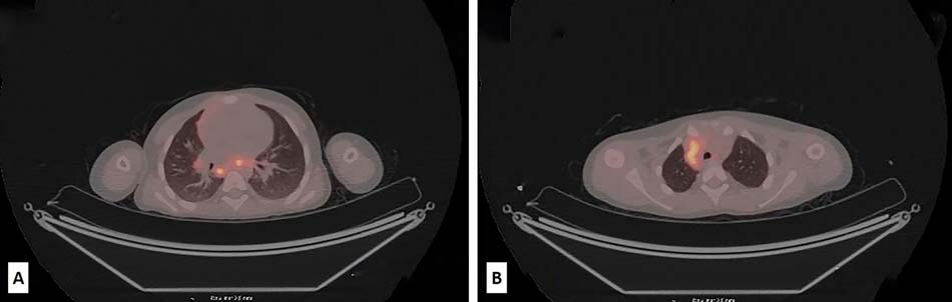
Low-dose PET/CT scan from the level of upper thoracic aperture revealing enlarged and metabolically active retrocaval and mediastinal lymph nodes, with SUV_max_ 8.6 and SUV_max_ up to 6.5 (A). Also, at the level of the carina and subcarinal region metabolically active lymph nodes were seen (SUV_max_ up to 7.6). In the right hilus, metabolically active lymph node SUV_max_ up to 3.5 was also observed (B).

The postoperative course was eventful. Four months later, during the routine follow-up, an abdominal CT scan revealed no residual disease but showed significant lymphadenopathy in the lower abdomen, while PET/CT revealed metabolically active deposits in the retroperitoneal, mediastinal, hilar and neck lymph nodes (Fig. 3A−B). A few suspected small nodes were also found in the left lower lung lobe. Lab tests showed high levels of LDH 548 U/L (range 100–190). Based on the clinical findings and the limited therapeutic options, the tumor board decided to do comprehensive genomic profiling of the tumor. The profiling was performed with the Oncomine Comprehensive Assay Plus (DNA, 498 genes) and Archer Fusion Plex Pan Solid Tumor v2 Panel (RNA, 137 genes) at the University Hospital Basel (Switzerland) (see Acknowledgement).

The genomic tests revealed a microsatellite stable tumor, without major driving or targetable mutations including *BAP1*, with no fusions, but with amplifications of *AURKA, AURKC, HLA-1B, ZNF-217, OR5F1* and *MEN1* genes. The tumor had a low mutational burden (1.9 mutations/Mb). Given the results of molecular profiling, the tumor board decided to treat the patient with adjuvant chemotherapy. However, during that period, the patient developed pneumonia and died of it at another hospital. A post-mortem examination was not performed.

## Discussion

Pediatric peritoneal mesotheliomas are very rare malignancies with limited data in the current literature. According to epidemiologic studies and autopsy results, mesotheliomas of children represent 2–5% of all mesotheliomas, with a reported incidence of 0.5–1 case/10 million per year [3]. These cancers may have some distinct clinicopathological features compared with their adult counterparts. There is a striking male-to-female predominance in adults (5:1), but in the pediatric population, mesotheliomas have nearly equal gender distribution. Also, there is a nearly equal distribution of peritoneal and pleural mesotheliomas in children compared to adults where pleural mesotheliomas predominate. Other characteristics, including age, the lack of previous asbestos or other carcinogen exposures, negative family history, and the presence of epithelioid morphology, with retained *BAP1* expression, are also known clinicopathologic features common in pediatric mesotheliomas [6]. The pathogenesis of pediatric mesotheliomas remains unclear, compared to adult mesotheliomas. Pediatric mesotheliomas are found to harbor different genetic abnormalities, which are not related to asbestos exposure [5]. Most of these characteristics were confirmed in our case [7–14].

There are no unified treatment protocols for peritoneal mesotheliomas, but cytoreductive surgery along with hyperthermic intraperitoneal preoperative surgery is considered the best treatment option. Our patient had surgery and was a candidate for adjuvant chemotherapy and eventually for targeted therapy.

Overall, malignant peritoneal mesothelioma has a poor prognosis, with a 10-year survival of only 9% [15, 16]. Limited targeted therapeutic options are available for these patients. Therefore, we conducted a comprehensive genomic profiling of our case, which revealed no somatic mutations including the *BAP1* gene (mutated in 85% of peritoneal mesotheliomas and around 30% of pleural mesotheliomas) [17], but amplifications of *AURKA, AURKC, HLA-1B, ZNF-217, OR5F1* and *MEN1* gene. Notably, the lack of *BAP1* genomic alterations correlated well with its retained protein expression by immunohistochemistry. Our comprehensive literature search on molecular profiling of pediatric peritoneal mesotheliomas revealed only a few studies (summarized in Table 1). The most prevalent genomic alterations included *ALK, STRN, TMP1, ATM* and *TERT* mutations, fusions, and rearrangements, with few chromosomal aberrations [18–24]. *ALK* gene alterations appear to be the most consistent genomic alterations in pediatric peritoneal mesotheliomas (Table 1). Notably, Murumägi *et al.* found the *STRK-ALK* rearrangement in disseminated peritoneal mesothelioma from a 5-year-old boy [19]. The patient was subsequently treated with an ALK inhibitor, reaching full remission for 3 years [19]. Several other genomic alterations have also been described in pediatric cases, including *ESWR1-ATF1* fusions and *ALK* rearrangements [23, 24] (Table 1). However, none of these genomic alterations were found in our case. The reasons for the discrepancies may be related to different genomic platforms for the assessment and panels used for the detection (Table 1).

**Table 1:** Overview of genomic features of peritoneal mesotheliomas in the pediatric population

**Year**	**Author**	**Patients (n)**	**Age (range)**	**Location**	**Molecular assay (method)**	**Identified genomic alterations (*n*)**
2021	Argani *et al.* [18]	5	15 years (mean) (range, 8–16)	Peritoneum and tunica vaginalis testis	FISH, Archer FusionPlex (RNA sequencing) and conventional cytogenetics	*STRN-ALK* fusion (*n* = 1), *ALK-TPM1* (*n* = 1) *ALK* rearrangements (*n* = 2) *t*(2;15) (*n* = 1)
2021	Murumagi *et al.* [19]	1	5 years	Peritoneum	NGS Foundation One, FISH	*STRN-ALK* fusion
2021	Sakata *et al.* [21]	1	14 years	Peritoneum	NGS	*STRN-ALK* rearrangement
2021	Olmedilla *et al.* [22]	1	12 years	Peritoneum	FISH, NGS (Impact panel)	*TERT* promoter translocation
2021	Ren *et al.* [23]	2	15 years both	Peritoneum and pericardium	RNA sequencing (Illumina TruSight RNA Fusion Panel), FISH	*EWSR1-ATF* fusion (n = 1) *EWSR1* rearrangement (*n* = 1)
2018	Mijalovsky *et al.* [20]	1	4 months	Peritoneum	WES	*ATM mutation*
2016	Loharamtaweethong *et al.* [32]	1	10 years	Peritoneum	FISH	*ALK* translocation
2013	Sugalski *et al.* [10]	1	7 years	Peritoneum	Conventional cytogenetic analysis	trisomy 11, deletion 19q13.33

FISH, Fluorescent In Situ Hybridization; NGS, next-generation sequencing; WES, whole exome sequencing.

Much more information on genomic features is available in adult mesotheliomas. Thus, Joseph *et al.* explored the mutational profile of 13 peritoneal mesotheliomas by NGS. They found the most frequent alteration to be the inactivation of the *BAP1* gene, followed by mutations in *NF2, SETD2* and *DDX3X*, homozygous deletion of *CDKN2,* and *MET* gene amplification [17]. Offin *et al.* reported similar genomic alterations among 50 adult peritoneal mesotheliomas (mutations of *BAP1, NF2, SETD2* and *TP53*) [25]. Takeda *et al.* performed FISH analysis in 54 cases and reported inconsistent and heterogeneous molecular genetic pathways in pleural and peritoneal mesotheliomas [26]. Similar to our case, Takeda *et al.* also found a predominantly low tumor mutational burden with a median number of mutations of 1.8/Mb [26]. Hung *et al.* reported additional genomic alterations among adult peritoneal mesotheliomas beyond *BAP1* mutations and *ALK* rearrangements, including *TP53, TFAF7* and *SUZ1* mutations [27].

One of the interesting findings from our case is *AURKA* gene amplification. Notably, Guo *et al.* found that the expression in Aurora kinase A (encoded by the *AURKA* gene) was significantly higher in malignant mesothelioma than in normal mesothelial cells and identified Aurora A as a potential diagnostic marker in differentiating benign mesothelial proliferation from malignant mesotheliomas [28]. Also, *AURKA* gene alterations were associated with poor prognosis [28]. The *AURKA* gene could be a potential therapeutic target with AURKA kinase A inhibitors [28]. Another relevant finding in our study is the Zinc finger protein 217 (*ZNF217*) gene amplification. ZNF217 is a transcription factor, involved in cancer initiation and progression [29]. Interestingly, Ugurluer *et al.* detected *ZNF217* mutation as a variant of unknown or additional significance in a small cohort of 11 pleural and peritoneal mesotheliomas [30]. Mutation in the *MEN1* gene could be related to Multiple endocrine neoplasia syndrome, while *OR5F1* alterations have not been reported yet in peritoneal mesotheliomas. Expression of HLA antigens can determine tumor progression and metastatic potential by influencing the immune response [31].

In conclusion, peritoneal mesothelioma in the pediatric population is extremely rare. It is still a fatal and aggressive disease with a poor outcome. Comprehensive genomic profiling may reveal novel therapeutic targets and should be done in such difficult-to-treat malignancies.

## Data Availability

Data available from the corresponding authors on a reasonable request.
